# *Gardenia jasminoides* Enhances CDDP-Induced Apoptosis of Glioblastoma Cells via AKT/mTOR Pathway While Protecting Death of Astrocytes

**DOI:** 10.3390/nu12010196

**Published:** 2020-01-10

**Authors:** Hyo In Kim, Se Hyang Hong, Jin Mo Ku, Min Jeong Kim, Sung Wan Ju, Seok Won Chang, Chunhoo Cheon, Seong-Gyu Ko

**Affiliations:** 1 Department of Science in Korean Medicine, College of Korean Medicine, Graduate School, Kyung Hee University, 26, Kyungheedae-ro, Dongdaemun-gu, Seoul 02447, Korea; hi9265@nate.com (H.I.K.); jung8328@hanmail.net (M.J.K.); seonca@naver.com (S.W.J.); 2 Department of Preventive Medicine, College of Korean Medicine, Kyung Hee University, 26, Kyungheedae-ro, Dongdaemun-gu, Seoul 02447, Korea; sehyang@khu.ac.kr (S.H.H.); saory_ykm@naver.com (J.M.K.); 3 Department of Korean Medicine, College of Korean Medicine, Graduate School, Kyung Hee University, 26, Kyungheedae-ro, Dongdaemun-gu, Seoul 02447, Korea; jsw2333@hanmail.net

**Keywords:** glioblastoma, *Gardenia jasminoides*, cisplatin (CDDP), synergy, apoptosis, autophagy, AKT/mTOR pathway

## Abstract

Gliomas are the most observed primary brain tumor, of which glioblastoma multiform (GBM) shows the highest incidence. Radiotherapy with temozolomide is the standard therapeutic method, but because of side effects, search for alternative therapies is required. *Gardenia jasminoides* (GJ) is flavonoid abundant with beneficial effects on inflammation, metabolic diseases, and cancers. In this study, we investigated the synergistic combination of GJ and cisplatin (CDDP) in U87MG and U373MG GBM cells. GJ and CDDP both showed cytotoxicity in U87MG cells, however GJ did not affect viability of normal astrocytes while CDDP displayed high toxicity. Cytotoxic effect of GJ and CDDP was related in apoptosis when confirmed by Western blot assays on cleaved caspase-3, caspase-9, and PARP. Moreover, GJ and CDDP showed synergistic combination in cell death of GBM cells, which was further confirmed by Western blot assays of apoptosis factors and also flow cytometry of Annexin V. Analysis on autophagy factors showed that GJ/CDDP combination induced autophagy, and through inhibition of autophagy, we could confirm autophagy is crucial to cytotoxicity of GJ/CDDP in GBM cell lines. The autophagy-mediated apoptosis of GJ/CDDP was dependent on the AKT/mTOR pathway. Overall, our results suggest GJ/CDDP combination as an effective yet safe therapeutic approach to GBMs.

## 1. Introduction

Gliomas are the most commonly observed primary brain tumor in humans. They account for 27% of all brain tumors and 80% of malignant brain [1], with incidence rates from 0.59 to 3.69 per 100,000 persons [2]. Glioma is a heterogeneous disease with multiple subtypes. Among the various types of gliomas, WHO grade IV glioblastoma multiform (GBM) is the one with the highest incidence (up to 45%) and displays the most aggressiveness [3].

Glioma treatment is considered one of the most difficult challenges for practitioners [4]. Guideline treatment for gliomas involves radiotherapy and chemotherapy after surgical intervention [5], which leads to age-standardized 10-year relative survival rate of low grade gliomas to be approximately 47% [6], however, in the case of GBM, of which the extensive tumor infiltration into the surrounding brain parenchyma makes surgery ineffective [7], the 5-year survival rate is less than 5%, with the median survival is only 14.6 months under therapy [8].

Although radiotherapy combined with temozolomide (TMZ) is mostly selected as the standard therapeutic method [9], however, due to the causable side effects from radiation, development of alternative therapies may lead to the next generation of GBM treatment. In line, cisplatin (CDDP), one of the most efficient antitumor drugs, is often chosen for an alternative chemotherapy agent of malignant gliomas. GBM usually harbors a wildtype *TP53* gene, and the rapid proliferation and resistance to cytotoxic treatment for GBM is attributed to the loss of p53 functions by post-translational modification [10,11]. The main mechanism of action of CDDP, induction of apoptosis by increasing p53 [12], leads to a logical selection of CDDP as a baseline therapy. Several studies report the potentially successful application of CDDP in gliomas experimentally [13,14,15], and clinically [16,17]. A recent study also reports that CDDP induces apoptosis by regulating autophagy in GBM cells [18].

*Gardenia jasminoides* (GJ) is a medicinal herb abundant with flavonoids [19,20], mainly used to treat inflammatory diseases, specifically jaundice and hepatitis in traditional Oriental medicine [21]. Besides its traditional use, studies have demonstrated GJ has beneficial effects on cardiovascular diseases [22], obesity [23], and various types of cancers [24,25], while protecting neuronal damage and cognitive deficits [26,27,28,29,30]. In this study, as combination therapy with cisplatin is frequently used to treat various types of cancers including brain tumor [31], we attempt to validate the synergistic effect of combination therapy of GJ and CDDP in U87MG and U373MG GBM cells.

## 2. Materials and Methods

### 2.1. Reagents

GJ powder provided by Hanpoong Pharmaceutical Co. (Jeonju, Korea) was dissolved in D.W. LY294002, SC79, 3-methyladenine (3-MA), 3-[4,5-dimetylthiazol-2-yl]-2,5-diphenyltetrazoliumbromide (MTT) and cisplatin were purchased from Sigma-Aldrich (St. Louis, MO, USA), and chloroquine (CQ) was from Invitrogen (San Diego, CA, USA). ECL solution were obtained from Merck Millipore (Middlesex, MA, USA) and Z-VAD-FMK was provided by R&D Systems, Inc. (Northeast, MN, USA), Dulbecco’s phosphate-buffered saline (DPBS), Dulbecco’s modified Eagle medium (DMEM), Roswell Park Memorial Institute 1640 (RPMI1640), penicillin and streptomycin were obtained from WELGENE (Gyeongsan, Korea). Fetal bovine serum (FBS) was obtained from GR scientific (Bedford, UK).

### 2.2. Cell Culture

Human glioblastoma cell line U87MG cells, U373MG cells, and normal astrocyte cells were obtained from the Korean Cell Line Bank of Seoul National University (Seoul, Korea). The U87MG cells and astrocyte cells were cultured in DMEM medium and U373 cells were cultured in RPMI1640 medium at 37 °C and 5% CO_2_. All media was supplemented with 10% FBS and 1% penicillin-streptomycin.

### 2.3. MTT Assay

Cell were seeded in a 96-well plate (1 × 10^4^ cells/well), incubated overnight, treated with GJ, CDDP, or GJ/CDDP for 24 h, and an MTT assay was performed as described previously [32].

### 2.4. Cell Morphology Observation and Crystal Violet Staining

Cells were seeded in a 6-well plate (1 × 10^6^ cells/well) and treated with GJ, CDDP, and GJ/CDDP for 24 h. After the culture media was discarded, the cells were washed with PBS for three times and were stained using crystal violet dye (Sigma-Aldrich, St. Louis, MO, USA) according to the instruction provided by the manufacturer. Representative pictures were taken under a regular light microscope to compare the growth of U87MG, U373MG cells, and astrocytes.

### 2.5. Combination Index (CI) Calculation

Compusyn ver. 1.0 (ComboSyn, Inc., Paramus, NJ, USA) was used according to the manufacturer’s instructions. Ratio of CDDP (µM) and GJ (µg/mL) was fixed to 1:250 and 1:500, and effect of four different combinations (1:250, 2:500, 1:500, 1:1000) was analyzed to determine the synergism between GJ and CDDP. Calculation formula for CI was as following [33]:

$$CI=\left(\frac{IC50\;of\;A\;in\;combination\;test}{IC50\;of\;A\;in\;single\;drug\;test}\right)+\left(\frac{IC50\;of\;B\;in\;combination\;test}{IC50\;of\;B\;in\;single\;drug\;test}\right)$$

### 2.6. Western Blot Assay

Western blot assay was performed as described previously [32]. Briefly, samples were loaded (20 µg) and electrophoresis was carried out at 60 V throughout stacking gel and at 120 V afterwards. Transfer to a polyvinylidene difluoride (PVDF) membrane was performed at 80 V for 2 h followed by blocking with 5% skimmed milk for 1 h. The membrane was incubated with following primary antibodies overnight in 4 °C: cleaved caspase-3 (Asp175) (#9661), cleaved caspase-9 (Asp330) (#9501), poly (ADP-ribose) polymerase (PARP) (#9542), SQSTM1/p62 (#5114), microtubule-associated protein 1A/1B-light chain 3 (LC3B) (#2775), p53 (#2527), Akt (#9272), p-Akt (#9271), ribosomal protein P70S6 kinase beta-1 (P70S6K), p-p70S6K and anti-glyceraldehyde-3-phosphate dehydrogenase (GAPDH) (#2118), all purchased from Cell Signaling technology (Danvers, MA, USA). Then, the membrane was incubated with appropriate secondary antibodies (1:1000) for 1 h, and enhanced chemiluminescence (ECL) solution was used to detect protein bands.

### 2.7. Flow Cytometry Assay

A flow cytometry assay was performed using BD FACScan System (BD Biosciences, San Jose, CA, USA) with Annexin-V-FITC (BD Biosciences, San Jose, CA, USA) and 7-aminoactinomycin D (7-AAD) (Sigma-Aldrich, St. Louis, MO, USA), as described previously [34]. Briefly, U87MG cells were seeded (1 × 10^5^ cells) in a 60-mm dish and treated with GJ, CDDP, or GJ/CDDP and incubated at 37 °C, 5% CO_2_ in 24 h. After collecting and washing the cells with PBS, the cells were stained with Annexin-V-FITC and 7-AAD to analyze apoptotic cell death by flow cytometry.

### 2.8. Statistical Analysis

Results are expressed as the mean ± standard error (S.E.) of three or more experiments. Statistical analyses were performed using GraphPad Prism 5 (GraphPad Software, Inc., La Jolla, CA, USA) by one-way ANOVA followed by a Tukey test to determine statistical differences (*p* < 0.05) between groups.

## 3. Results

### 3.1. GJ and CDDP Treatment Induce Cell Death of U87MG and U373MG Cells

**Figure 1:**
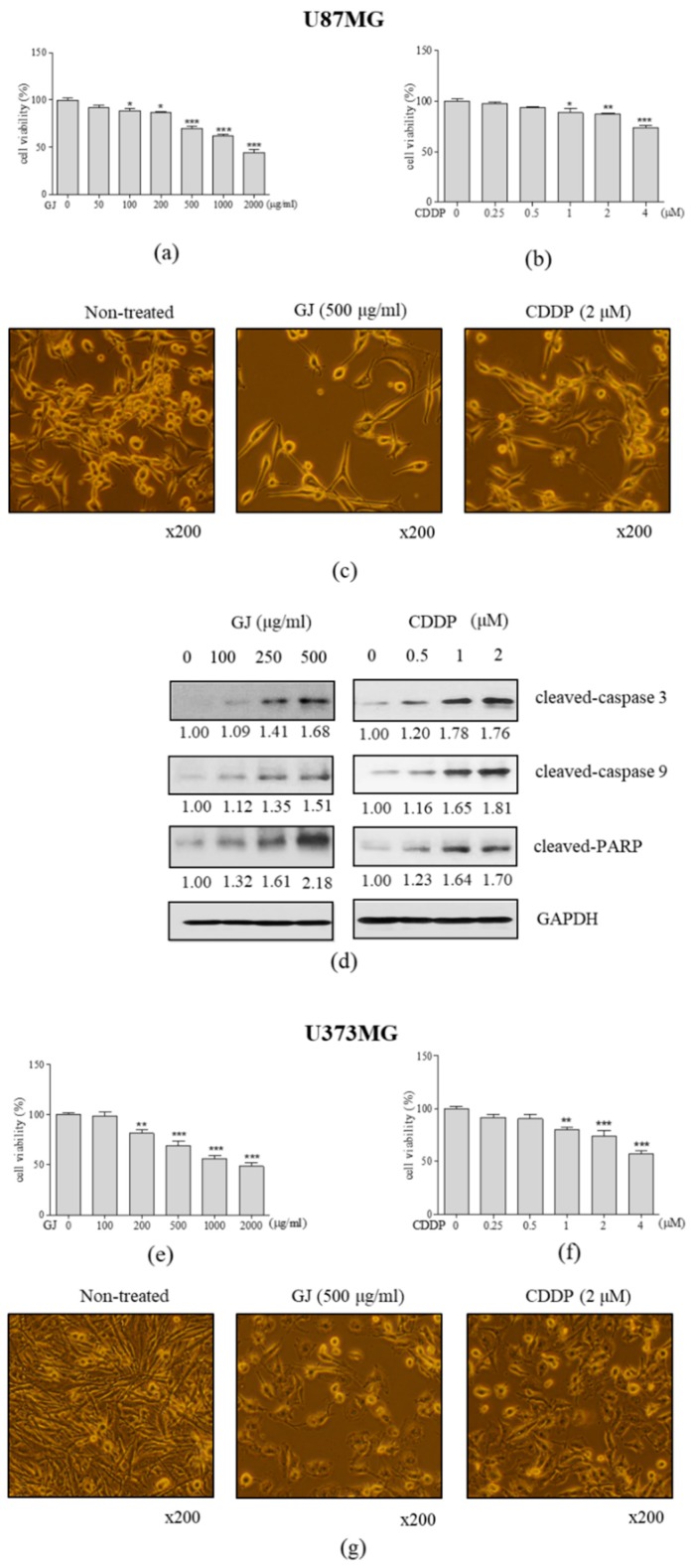
Effect of GJ and CDDP on cell viability of U87MG and U373MG cells. MTT assays were performed in (**a**) GJ-treated and (**b**) CDDP-treated U87MG cells. (**c**) Microscopical observation of cell morphology was performed in GJ- and CDDP-treated U87MG cells. (**d**) Western blot assays on cleaved caspase-3, cleaved caspase-9, and cleaved poly (ADP-ribose) polymerase (PARP) were performed in GJ- and CDDP-treated U87MG cells. MTT assays were performed in (**e**) GJ-treated and (**f**) CDDP-treated U373MG cells. (**g**) Microscopical observation of cell morphology was performed in GJ- and CDDP-treated U373MG cells. Results are displayed as mean ± S.E. of three or more separate experiments. Glyceraldehyde-3-phosphate dehydrogenase (GAPDH) was used as endogenous control. * *p* < 0.05 vs. untreated cells, ** *p* < 0.01 vs. untreated cells, *** *p* < 0.005 vs. untreated cells. GJ, *Gardenia jasminoides*; CDDP, cisplatin.

To establish the effective concentration of GJ and CDDP, we performed a cytotoxicity test of MTT in U87MG GBM cell line and normal astrocytes. When U87MG cells were treated with GJ for 24 h, significant inhibition of cell viability was displayed in concentrations over 100 µg/mL with IC50 between 1000 and 2000 µg/mL (Figure 1a), of which concentrations showed less than 20% of cytotoxicity in normal astrocytes (Figure S1a). Decrease of cell viability were shown in U373MG cell line similar to that of U87MG cells (Figure 1e). However, on the other hand, CDDP treatment showed significant cytotoxicity in U87MG and U373MG cells at 1 µM (Figure 1b,f), which induced nearly 50% of cell death of astrocytes (Figure S1b). By confirming through observation on cell morphology (Figure 1c,g), GJ treatment (500 µg/mL) showed higher cytotoxic effect in GBM cells than CDDP (2 µM). In contrast, GJ did not seem to affect cell viability of normal astrocytes while CDDP induced observational cell death (Figure S1c).

Next, we confirmed the effect of both treatments on apoptotic factors by Western blot. As a result, we observed dose-dependent increase of cleaved-caspase 3, cleaved-caspase 9, and cleaved-PARP in both GJ- and CDDP-treated GBM cells (Figure 1d).

### 3.2. GJ/CDDP Combination Synergistically Induces Cell Death of U87MG and U373MG Cells

**Figure 2:**
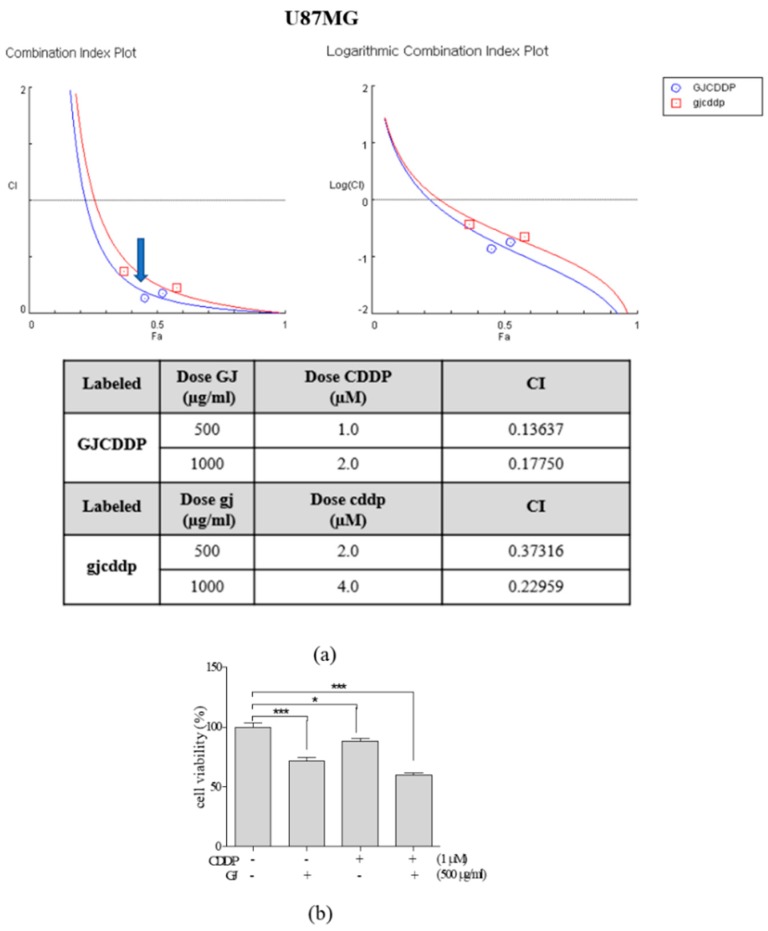

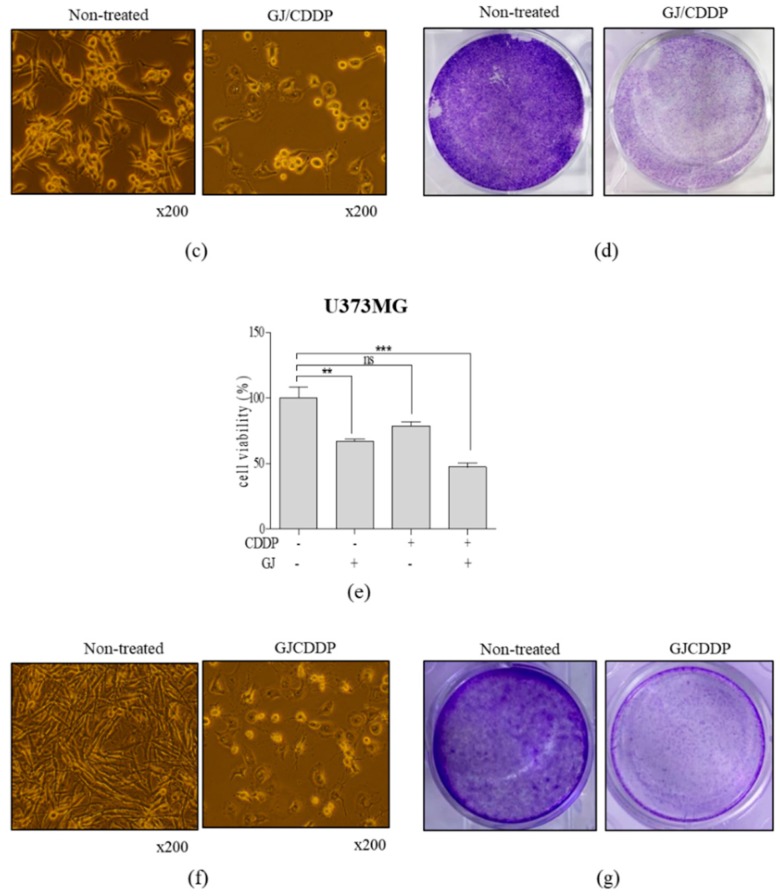
Synergistic evaluation of GJ/CDDP combination on cytotoxicity in U87MG and U373MG cells. (**a**) Combination index (CI) (left) and logarithmic CI (right) of GJ/CDDP combination was evaluated. (**b**) An MTT assay was performed to compare the cell viability in GJ-, CDDP-, and GJ/CDDP-treated U87MG cells. (**c**) Microscopic observation of cell morphology in GJ/CDDP combination-treated U87MG cells was performed. (**d**) Crystal violet staining was performed in GJ/CDDP combination-treated U87MG cells. (**e**) An MTT assay was performed to compare the cell viability in GJ-, CDDP-, and GJ/CDDP-treated U373MG cells. (**f**) Microscopic observation of cell morphology in GJ/CDDP combination-treated U373MG cells was performed. (**g**) Crystal violet staining was performed in GJ/CDDP combination-treated U373MG cells. Results are displayed as mean ± S.E. of three or more separate experiments. * *p* < 0.05 vs. untreated cells, ** *p* < 0.01 vs. untreated cells, *** *p* < 0.005 vs. untreated cells. GJ, *Gardenia jasminoides*; CDDP, cisplatin.

Based on the above results, we then assessed the synergistic effect of GJ and CDDP when their combination was treated in U87MG GBM cells. We established two different combination ratios (250:1 and 500:1 (µg/mL:µM)) of GJ:CDDP and calculated CI of the GJ/CDDP combination (Figure 2a). As CI of 500:1 was relatively lower than 250:1, we selected 500:1 as the combination ratio of GJ/CDDP for further experiments. As shown in Figure 2b, the combination of GJ and CDDP showed significant synergism in cell viability of U87MG cells. GJ/CDDP combination showed higher cytotoxicity compared to single treatments of either GJ or CDDP in U373MG cells as well (Figure 2e). On the other hand, GJ/CDDP combination showed less effect in cell death of normal astrocytes, suggesting the supplementation of GJ improved CDDP-induced cytotoxicity in astrocytes (Figure S2).

Further observations were performed in GJ/CDDP combination-treated U87MG cells to verify the MTT results. GJ/CDDP treatment reduced the number of live cells both confirmed by microscopic observation of cell morphology (Figure 2c,f) and crystal violet staining of live cells (Figure 2d,g).

### 3.3. GJ/CDDP Combination Induces Apoptosis in U87MG and U373MG Cells

**Figure 3:**
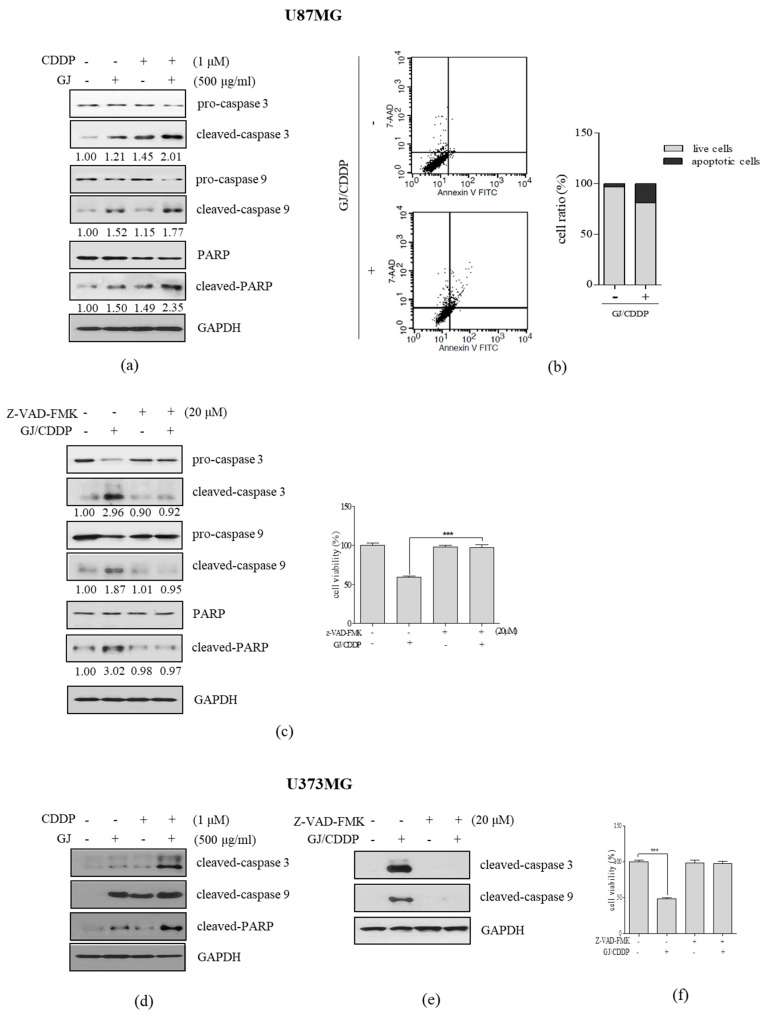
Effect of GJ/CDDP combination on apoptosis factors in U87MG and U373MG cells. (**a**) Western blot assays on cleaved caspase-3, cleaved caspase-9, and cleaved PARP were performed in GJ/CDDP combination-treated U87MG cells. (**b**) Annexin V assay was performed in GJ/CDDP combination-treated U87MG cells. (**c**) An MTT assay was performed in GJ/CDDP combination-treated U87MG cells after pre-treatment with Z-VAD-FMK. (**d**) Western blot assays on cleaved caspase-3, cleaved caspase-9, and cleaved PARP were performed in GJ/CDDP combination-treated U373MG cells. (**e**,**f**) An MTT assay was performed in GJ/CDDP combination-treated U373MG cells after pre-treatment with Z-VAD-FMK. Results are displayed as mean ± S.E. of three or more separate experiments. GAPDH was used as endogenous control. *** *p* < 0.005 vs. untreated cells. GJ, *Gardenia jasminoides*; CDDP, cisplatin.

Next, to evaluate whether GJ/CDDP treatment induced apoptosis in GBM cells, we measured the protein expression of crucial apoptotic factors including cleaved-caspase 3, 9, and cleaved PARP. Compared to GJ (1.21-, 1.52-, 1.50-fold increase in cleaved-caspase 3, cleaved-caspase 9, and cleaved-PARP, respectively) and CDDP (1.45-, 1.15-, 1.49-fold increase in cleaved-caspase 3, cleaved-caspase 9, and cleaved-PARP, respectively), GJ/CDDP combination showed higher expressions in these factors of 2.01-, 1.77-, 2.35-fold increase in cleaved-caspase 3, cleaved-caspase 9, and cleaved-PARP, respectively (Figure 3a). In addition, assessing cell count after Annexin V staining by flow cytometry analysis showed increased apoptosis by GJ/CDDP treatment (Figure 3b). Further confirmation in MTT assays after Z-VAD-FMK treatment, a pan-caspase inhibitor, we found out that GJ/CDDP treatment cannot display cell toxicity in apoptosis-inhibited conditions (Figure 3c), suggesting that GJ/CDDP affects cell viability by increasing apoptosis of U87MG cells. Consistently, similar results were observed in U373MG cells (Figure 3d–f).

### 3.4. GJ/CDDP Combination-Induced Apoptosis in U87MG and U373MG Cells Is Dependent on Autophagy Pathway

**Figure 4:**
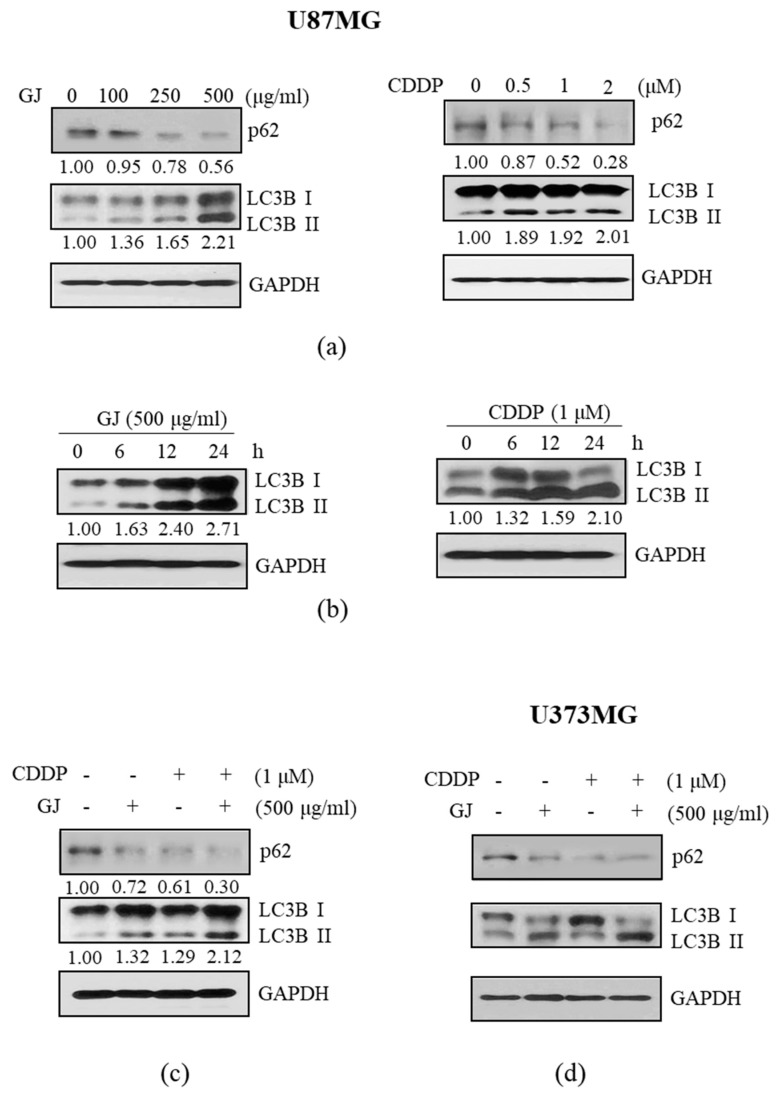
Effect of GJ/CDDP combination on autophagy factors in U87MG and U373MG cells. (**a**) Western blot assays on p672 and microtubule-associated protein 1A/1B-light chain 3 (LC3B)-I/II were performed in GJ- and CDDP-treated U87MG cells. (**b**) Time-dependent change of LC3B-I/II in GJ- and CDDP-treated U87MG cells were measured by Western blot assays. (**c**) Effect of GJ/CDDP combination on p62 and LC3B-I/II was compared to GJ and CDDP single treatment by Western blot assays. (**d**) Western blot assays on p62 and LC3B-I/II were performed in GJ- and CDDP-treated U373MG cells. GAPDH was used as endogenous control. GJ, *Gardenia jasminoides*; CDDP, cisplatin.

Based on a recent study by Ma et al. [18] reporting that CDDP-induced apoptosis in U87 cells is dependent on autophagy, we investigated whether this pathway also corresponds to the effect of GJ/CDDP combination. First, proteins which have crucial roles in the autophagic flux was investigated. As in Figure 4a,d, we could observe dose-dependent inhibition of p62 and induction of LC3B-II in GBM cell lines by both GJ and CDDP treatment. The time-dependent regulation of autophagosome formation by these two treatments was also verified (Figure 4b). Further, Western blot assay was performed to confirm the effect of GJ/CDDP combination on the autophagy pathway, to show the combination resulted in lower expression of p62 and higher expression of LC3B-II than single treatments of each (Figure 4c).

### 3.5. GJ/CDDP Combination-Induced Autophagy in U87MG and U373MG GBM Cells

**Figure 5:**
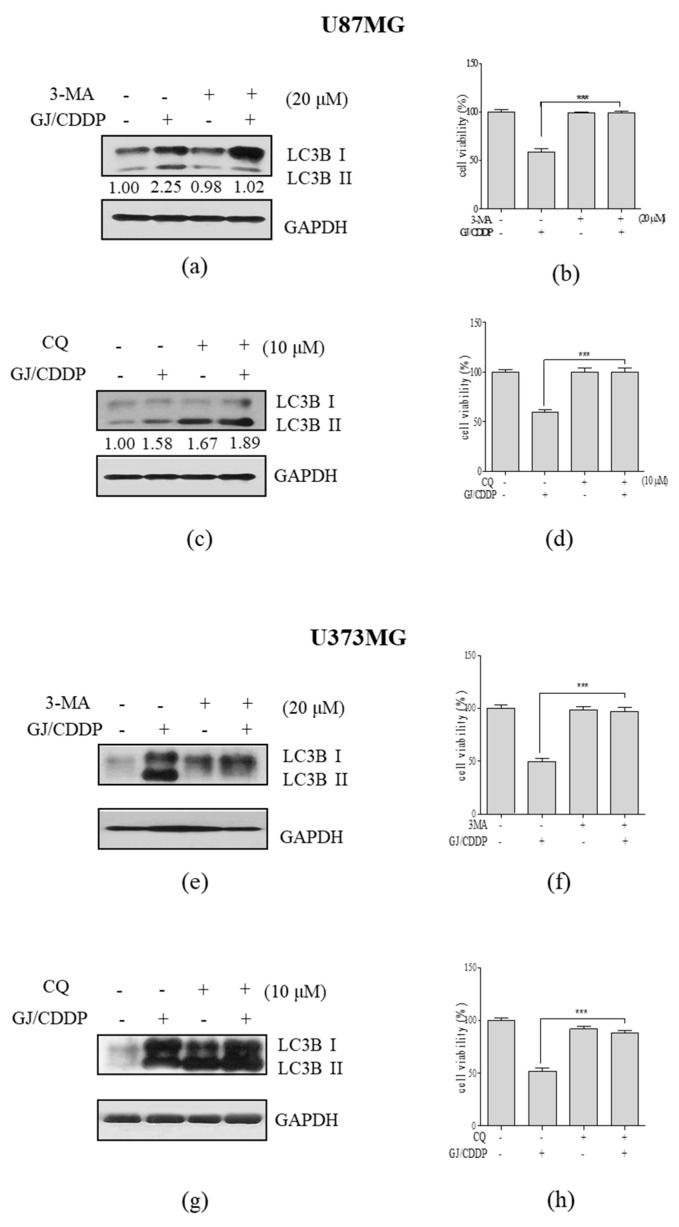
Effect of GJ/CDDP combination under autophagy-inhibited conditions in U87MG and U373MG cells. (**a**) A Western blot assay on LC3B-I/II and (**b**) an MTT assay was performed in GJ/CDDP combination-treated U87MG cells after pre-treatment with 3-MA. (**c**) A Western blot assay on LC3B-I/II and (**d**) an MTT assay was performed in GJ/CDDP combination-treated U87MG cells after pre-treatment with CQ. (**e**) A Western blot assay on LC3B-I/II and (**f**) an MTT assay was performed in GJ/CDDP combination-treated U373MG cells after pre-treatment with 3-MA. (**g**) A Western blot assay on LC3B-I/II and (**h**) an MTT assay was performed in GJ/CDDP combination-treated U373MG cells after pre-treatment with CQ. Results are displayed as mean ± S.E. of three or more separate experiments. GAPDH was used as endogenous control. *** *p* < 0.005 vs. untreated cells. GJ, *Gardenia jasminoides*; CDDP, cisplatin.

To verify the role of autophagy in GJ/CDDP-induced cell death, we pre-treated U87MG cells and U373MG cells with 3-MA, the early stage inhibitor of autophagy, and CQ, the late stage inhibitor of autophagy. When pre-treated with 3-MA, increase of LC3B-II by GJ/CDDP was inhibited (Figure 5a,e), and MTT assay showed decrease in cytotoxicity (Figure 5b,f) implying the blockage of autophagosome formation nullifies the effect of GJ/CDDP. Pre-treatment with CQ showed a massive 1.89-fold increase by GJ/CDDP treatment compared to untreated cells, indicating the accumulation of autophagosomes (Figure 5c). GJ/CDDP combination also increased LC3B-II accumulation in U373MG GBM cells (Figure 5g). Moreover, GJ/CDDP-induced decrease of cell viability in both U87MG and U373MG cells was not observed when CQ was pre-treated (Figure 5d,h). From the above results, we could conclude the autophagic flux is crucial in the cytotoxic action of GJ/CDDP.

### 3.6. GJ/CDDP Combination Induces Autophagy-Dependent Apoptosis in U87MG and U373MG Glioblastoma Cells via PI3K/AKT Pathway

**Figure 6:**
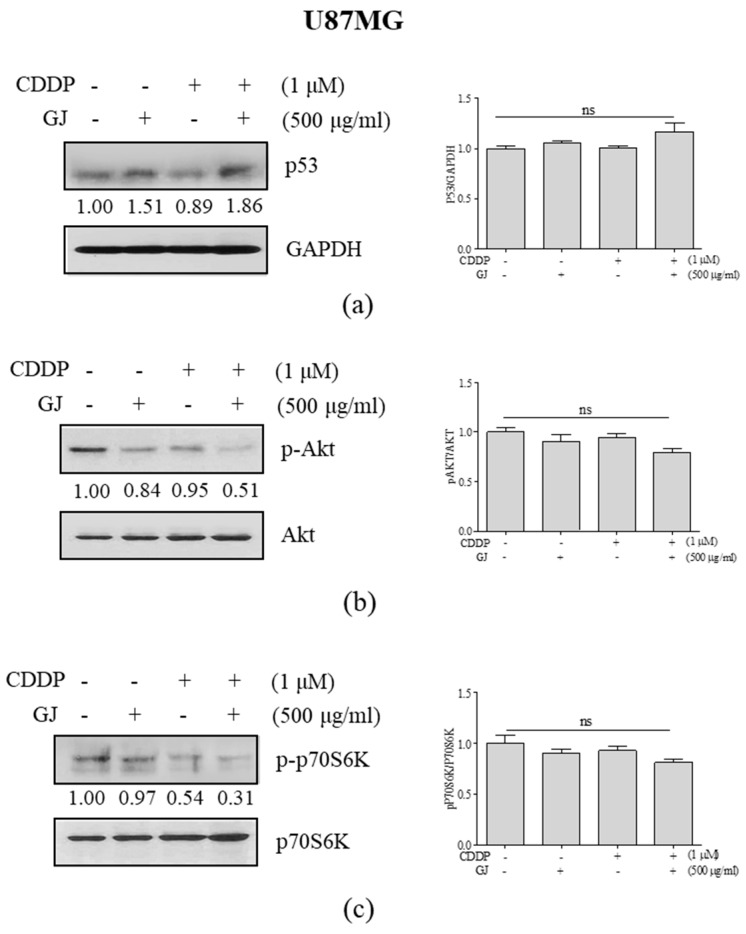

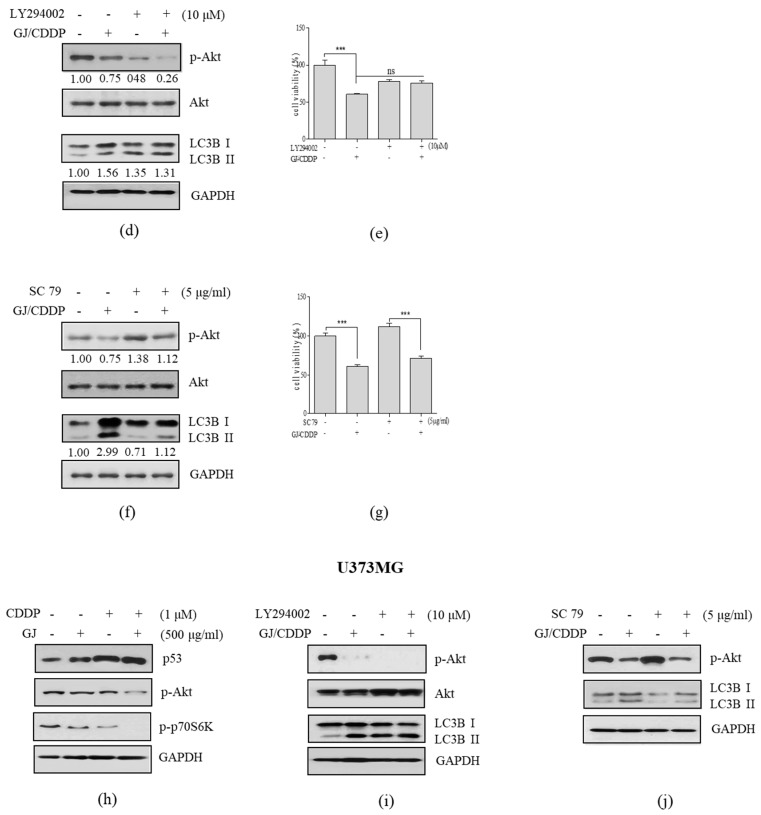
Effect of GJ/CDDP combination on AKT/mTOR pathway in U87MG and U373MG cells. Western blot assays on (**a**) p53, (**b**) p-AKT, and (**c**) p-p70S6K were performed in GJ/CDDP combination-treated U87MG cells. (**d**) Western blot assays on p-AKT and LC3B-I/II, and (**e**) an MTT assay were performed in GJ/CDDP combination-treated U87MG cells after pre-treatment with LY294002. (**f**) Western blot assays on p-AKT and LC3B-I/II, and (**g**) an MTT assay was performed in GJ/CDDP combination-treated U87MG cells after pre-treatment with SC79. (**h**) Western blot assays on p53, p-AKT, and p-p70S6K were performed in GJ/CDDP combination-treated U373MG cells. (**i**) Western blot assays on p-AKT and LC3B-I/II were performed in GJ/CDDP combination-treated U373MG cells after pre-treatment with LY294002. (**j**) Western blot assays on p-AKT and LC3B-I/II were performed in GJ/CDDP combination-treated U373MG cells after pre-treatment with SC79. Results are displayed as mean ± S.E. of three or more separate experiments. GAPDH was used as endogenous control. *** *p* < 0.005 vs. untreated cells. GJ, *Gardenia jasminoides*; CDDP, cisplatin.

The importance of TP53 regulation in GBM treatment is well-established [10,11], and the apoptotic effect of CDDP treatment is related to the increase of p53 [12]. Thus, we investigated the relevance of p53 and its downstream signaling pathways AKT/mTOR in the action of GJ/CDDP. When compared to GJ and CDDP single treatments, GJ/CDDP combination increased the expression of p53 (Figure 6a) and suppressed the expressions of p-AKT (Figure 6b) and p-p70S6K (Figure 6c). Increase of these factors in U373MG cells were also induced by GJ/CDDP co-treatment (Figure 6h).

To elucidate the role of AKT in the cytotoxic effect of GJ/CDDP, we then treated U87MG cells either with the PI3K inhibitor LY294002 and AKT activator SC79 in prior to GJ/CDDP treatment. As shown in Figure 6d, in a PI3K/AKT-inhibited condition, GJ/CDDP failed to induce autophagy, and also could not affect cell viability (Figure 6e). On the other hand, cell viability of GBM cells were increased when AKT phosphorylation was induced by SC79 treatment, however, GJ/CDDP co-treatment competitively reduced the SC79-induced cell viability (Figure 6g) by inhibiting p-AKT expression (Figure 6f). Further investigation in U373MG GBM cells with same methodology confirmed these results (Figure 6i,j). These results demonstrate that AKT/mTOR signaling plays a crucial role in the GJ/CDDP-mediated cell death of GBM cells.

## 4. Discussion

Brain and other central nervous system (CNS) cancer is the 10th leading cause of death. Almost 24,000 adults (13,410 men/10,410 women) in the United States are estimated to be diagnosed, and 18,000 adults (9910 men/7850 women) are estimated to die from primary brain and CNS cancer in the year 2019 according to the Central Brain Tumor Registry of the United States [1].

Craniotomy surgical resection followed by combination of radiotherapy and chemotherapy is still the standard therapeutic strategy for GBM. As an alkylating agent inducing cell cycle arrest [35], TMZ has been utilized for the therapeutic approach in GBM for decades. However, application of TMZ chemotherapy is not effective to at least 50% of GBM patients, because of either overexpressed O^6^-methylguanin methyltransferase, lack of DNA repairing pathway, or both can be present in GBM cells [36]. In this case, the alternative drug considered is cisplatin, as it can induce apoptosis via p53 pathway [12]. The tumor suppressor/transcription factor p53 plays a crucial role in tumor prevention by regulating numerous cellular responses, including apoptosis, angiogenesis, cancer cell metabolism, and tumor microenvironment [37]. Enforced expression of *TP53* impairs the growth of transformed cells, providing evidence that *TP53* functions as a tumor suppressor [38]. Furthermore, *TP53* is one of the most commonly deregulated genes in cancer. The mutation of TP53 is associated with progression of GBM [39] while inactivation of p53 inactivation is positively correlated to invasiveness [40] and proliferation [41], and negatively correlated to apoptosis [42] in GBM. According to The Cancer Genome Atlas project, the p53 pathway (including CDKN2A, MDM2 and TP53) was deregulated in ~85% of GBM patients [43]. In this study, we observed enhanced apoptosis in U87MG and U373MG cells by both GJ and CDDP treatment, and these two showed synergism when combined.

GJ is a widely used medicinal herb in traditional medicine of Oriental countries including China, Japan, and Korea. Applying the dried fruit of this plant from Rubiaceae family, practitioners attempt to reduce inflammatory symptoms which result from hepatic disorders. Experimental approaches reported its hepatoprotective [44], anti-inflammatory effects in diseases such as allergy [45,46,47], pancreatitis [48], and endothelial inflammation [49]. Furthermore, numerous reports provide evidence for the anti-cancer effects of GJ and its constituents. Suppressive effect on oral cancer [24] and melanoma [25] by GJ treatment is reported, and genipin is known to display anti-cancer effects in breast cancer [50], colorectal cancer [51,52,53], bladder cancer [54], liver cancer [55], and myeloma [56]. In gliomas, Wang and colleagues have published several studies through intense research regarding the anti-proliferative, cell cycle-inhibitory and apoptotic effect of penta-acetyl geniposide in glioma cells [57,58,59,60,61,62,63,64,65]. These studies provide evidence for the potentially beneficial use of GJ in GBMs, however, up to date, the synergistic effect between GJ and CDDP in GBMs has not been investigated.

In addition to the potential anti-cancer effect on GBM, evidence on the effects of GJ in the CNS suggests it may be beneficially act as a supplemental agent for GBM treatment. GJ inhibits LPS-induced inflammation in BV-2 microglia cells [27] and shows protective effects in amyloid beta peptide-induced neuron damage [26]. GJ also displays beneficial effects on cognitive deficits/impairments in Alzheimer’s animal models [28,29,30]. Further literature suggests active compounds derived from GJ such as genipin and geniposide possess similar neuroprotective effects. Genipin was reported to promote prominent neuronal growth in PC12 cells [66]. On the other hand, geniposide has neuroprotective effects in PC12 cells [67,68] and SH-SY5Y human neuroblastoma cell line [69]. It also improved side effects of fluoxetine, the serotonin reuptake inhibitor, by ameliorating its suppression effect on neurite differentiation [70]. In line, our results showed that CDDP treatment induced cell death in normal human astrocytes, but when combined with GJ, the cytotoxicity in normal astrocytes were improved while the cytotoxic effect in GBM cells were synergistically increased. In the use of combination with CDDP, a study by Mahgoub et al. may also suggest another beneficial use of GJ on a possible CDDP side effect in the kidney [71].

Besides induction of apoptosis, the anti-oncogenic signaling pathways triggered by known tumor-suppressor proteins including p53 may also stimulate autophagy [72]. The bi-effective role of autophagy in cancer offers a high potential for future therapy, and therefore is intensively investigated by researchers in the oncology field. In contrast from conventional programmed cell death (i.e., apoptosis and necrosis), induction of autophagy can lead to either a pro-survival or a pro-death strategy [72]. This falls into the treatment of GBM as well, thus the role of autophagy for the therapeutic approach in GBM is gaining interest [73]. Our current study investigated the beneficial effect of combination therapy of p53-regulating anti-cancer agent cisplatin with GJ, a natural product, in GBM, mainly focusing on the relevance of autophagic pathway. GJ/CDDP combination treatment showed enhanced autophagic flux, and by co-treatment of the pan-caspase inhibitor Z-VAD-FMK, we could conclude the apoptotic effect of GJ/CDDP combination was related to the induction of autophagy. To elucidate the underlying mechanism of this action, we investigated the AKT/mTOR pathway, the well-known pathway which regulates autophagy and also plays a key role in cell survival/death of gliomas [74]. Our results indicated that this pathway is crucial for the apoptotic action of GJ/CDDP combination in U87MG and U373MG GBM cells.

The existence of blood-brain barrier (BBB) is one of the major obstacles in GBM medication. Previous reports have conducted studies with GJ or its constituents to investigate whether they are able to cross the BBB. Lu et al. [75] reported that geniposide can be basically transported into the brain through blood vessels when used as Gardenia–Borneol co-compound. Yu et al. [76] also determined the delivery of geniposide to four brain regions in the absence/presence of borneol in rat, demonstrating that borneol observably promoted the delivery to hippocampus and hypothalamus, and Chen et al. [77] established a BBB-mimicking in vitro model using Madin-Darby canine kidney cells to show the transport of geniposide through BBB is enhanced by combination with borneol and muscone. Our current study showed GJ can enhance CDDP sensitivity and induce cell death of GBM, and literature supports its possible application by reporting the BBB-crossing of geniposide.

The translational relevance between in vitro and in vivo models has always been an issue in GBM research. In vitro models, compared to in vivo approaches, are tractable and cost effective. Recently developed patient-derived model provides further advantages. The patient-derived GBM cells are reported to be sustained longer that conventional GBM cell lines [78]. Furthermore, Lancaster et al. [79] have successfully built an organoid of human CNS from pluripotent stem cells, which will lead the in vitro GBM research to the next paradigm. However, despite the many advantages of in vitro approaches along with the effort to link cell line studies to clinical application, the cellular models of GBM indeed have limitations. The role of human immune system within the tumor microenvironment may be the main reason why whole-animal models undoubtedly provide key features which cannot be neglected. Further investigation is necessary to support our results showing that GJ and CDDP can synergistically increase autophagy-mediated apoptosis in GBMs. Patient-derived primary GBM cell culture model or GBM organoid models, even an in vivo study should be carried out to validate the efficacy of GJ in GBM treatment. However, although its clinical application still requires intensive investigation, yet the subculture of the “classic” GBM cell lines such as U87MG allows researchers to investigate the characteristics of GBM without the interference of extrinsic variables [80]- thus, the first step of GBM research should be departed by GBM cell line experiments, and our study will provide evidence for a potentially effective and safer pharmaceutical agent for GBM treatment.

## 5. Conclusions

In this study, we showed that GJ/CDDP combination exerts higher apoptotic potential compared to treatment of CDDP only. Furthermore, the cytotoxicity of CDDP is alleviated by the combination of GJ. The anti-cancer effect of GJ/CDDP treatment is dependent on the induction of autophagy, in which process the AKT/mTOR pathway plays a crucial role. In conclusion, we suggest GJ as a potential agent in combination with CDDP in GBM treatment, as it shows synergistic increase of cell death and also displays protective effect on unintended cell death of astrocytes caused by CDDP.

## Supplementary Materials

The following are available online at https://www.mdpi.com/2072-6643/12/1/196/s1. Figure S1. Effect of GJ and CDDP on cell viability of normal human astrocytes. Figure S2. GJ attenuates CDDP-induced cytotoxicity in normal human astrocytes.
