# Supplementary material for: Gardenia jasminoides Enhances CDDP-Induced Apoptosis of Glioblastoma Cells via AKT/mTOR Pathway While Protecting Death of Astrocytes
Source: Nutrients. 2020 Jan 10;12(1):196. doi: 10.3390/nu12010196 (PMC7019269; doi:10.3390/nu12010196)
Supplement: Supplementary file 1 [file nutrients-12-00196-s001.pdf]

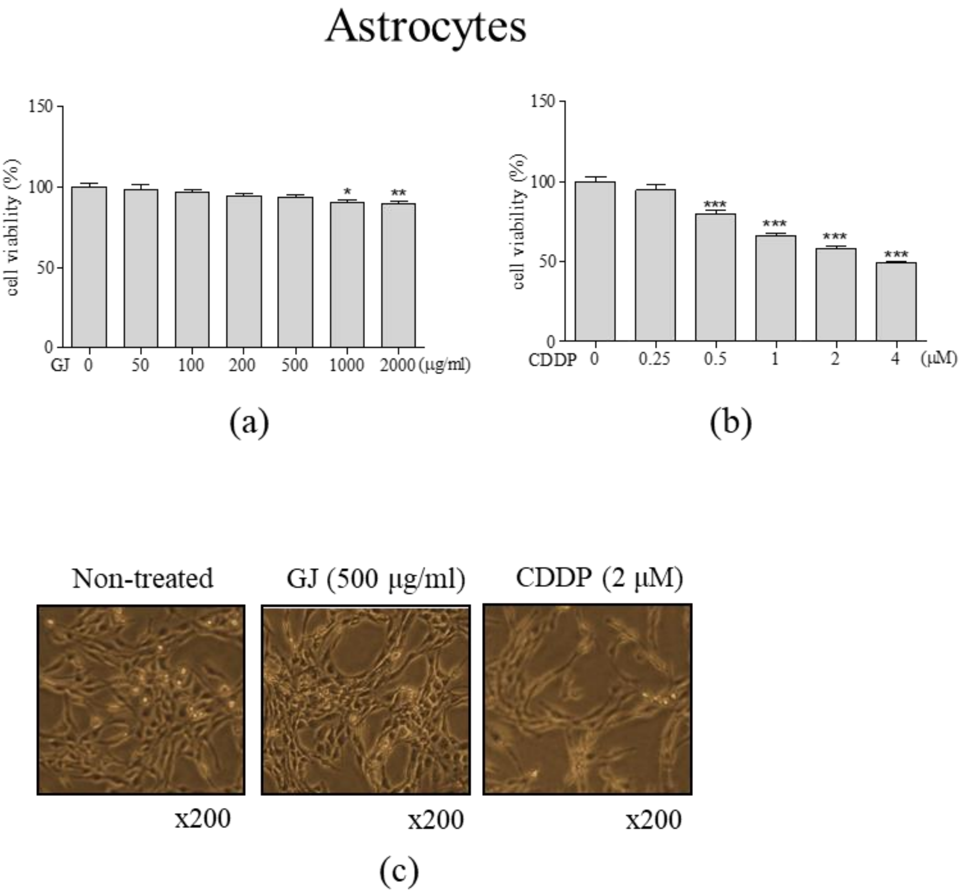

Figure S1. Effect of GJ and CDDP on cell viability of normal human astrocytes. MTT assays in (a) GJ-treated and (b) CDDP-treated normal human astrocytes were performed. (c) Microscopic observation in GJ- and CDDP-treated normal human astrocytes were performed. Results are displayed as mean ± S.E. of three or more separate experiments. *p < 0.05 vs. untreated cells, **p < 0.01 vs. untreated cells, ***p < 0.005 vs. untreated cells. GJ, *Gardenia jasminoides*; CDDP, cisplatin.

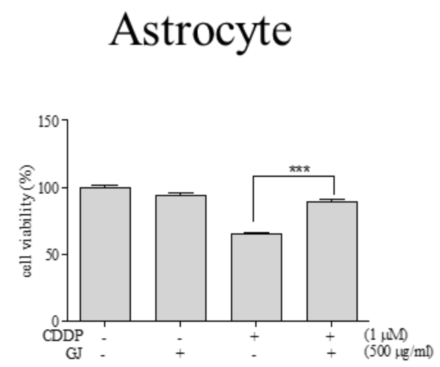

Figure S2. GJ attenuates CDDP-induced cytotoxicity in normal human astrocytes. Results are displayed as mean ± S.E. of three or more separate experiments ***p < 0.005. GJ, *Gardenia jasminoides*; CDDP, cisplatin.
